# Exploring bridge symptoms in HIV-positive people with comorbid depressive and anxiety disorders

**DOI:** 10.1186/s12888-022-04088-7

**Published:** 2022-07-05

**Authors:** Xiaoning Liu, Hui Wang, Zheng Zhu, Liyuan Zhang, Jing Cao, Lin Zhang, Hongli Yang, Huan Wen, Yan Hu, Congzhou Chen, Hongzhou Lu

**Affiliations:** 1 Department of Infectious Diseases, National Clinical Research Center for Infectious Diseases, Shenzhen Third People’s Hospital, Shenzhen, 518112 Guangdong Province China; 2 National Heart & Lung Institute, Faculty of Medicine, Imperial College London, London, UK; 3 School of Nursing, Fudan University, 305 Fenglin Rd, Shanghai, 200032 China; 4 Fudan University Centre for Evidence-based Nursing: A Joanna Briggs Institute Centre of Excellence, Shanghai, China; 5 Department of Nursing, Shanghai Public Health Clinical Center, Shanghai, China; 6 Department of Nursing, Yunnan Provincial Infectious Disease Hospital, Kunming, Yunnan China; 7 School of Public Health, Fudan University, Shanghai, China; 8 School of Public Health, Faculty of Medicine, Imperial College London, London, UK

**Keywords:** HIV/AIDS, Depression, Anxiety, Symptom network, Bridge symptom

## Abstract

**Background:**

The prevalence of comorbid depressive and anxiety disorders in people living with HIV (PLWH) is high. However, it is unclear which symptom is the bridge symptom between depression and anxiety in PLWH. This study aimed to develop symptom networks for depression and anxiety and explore the bridge symptoms and interconnectedness between these disorders in PLWH with comorbid depressive and anxiety disorders.

**Methods:**

A multisite, hospital-based cross-sectional study was conducted from March 2020 to November 2021. Depression and anxiety were measured with the Hospital Anxiety and Depression Scale. We visualized the symptom network using the *qgraph* package and computed the bridge expected influence of each node. The GLASSO layout was used to generate undirected association networks.

**Results:**

A total of 2016 individuals were included in the analysis. In the anxiety cluster, “not feeling relaxed” had the highest bridge expected influence and strength (*r*_*bridge expected influence*_ = 0.628, *r*_*strength*_ = 0.903). In the depression cluster, “not feeling cheerful” was identified as having a high bridge expected influence (*r*_*bridge expected influence*_ = 0.385). “Not feeling cheerful” and “not feeling relaxed” were the strongest edges across the depression and anxiety clusters (*r* = 0.30 ± 0.02).

**Conclusions:**

Healthcare professionals should take note when PLWH report severe bridge symptoms. To enhance the levels of perceived cheerfulness and relaxation, positive psychology interventions could be implemented.

## Introduction

Human Immunodeficiency Virus (HIV) is a major public health issue that had affected over 37 million people worldwide by the end of 2020 [1]. There is no cure for HIV. However, due to the widespread use of antiretroviral therapy (ART), HIV infection has been transformed from a fatal diagnosis into a lifelong manageable chronic disease [2]. People living with HIV (PLWH) face a series of mental health conditions, such as depression and anxiety. HIV-related and socioeconomic factors, including physical symptoms, ART side effects, economic and medical difficulties, social stigma and discrimination, and/or lack of social support, can all cause these mental problems [3, 4].

In PLWH, anxiety and depression are the most common mental disorders. PLWH are twice as likely as the general population to be diagnosed with depression [5]. The prevalence of global depression in PLWH was 31% based on screening instruments, which ranged from 22% in Europe to 44% in South America [6]. In addition, some low- and middle-income regions reported a prevalence of depression higher than 30% [7]. The prevalence of anxiety in PLWH varies due to different study designs and sample sizes; however, it is widely believed that the prevalence of anxiety ranges from approximately 19 to 49%, compared to a twelve-month prevalence of 18% and a lifetime prevalence of 29% in general population [8–10]. High severity of depression and anxiety can lead to low levels of ART adherence and health-related quality of life, treatment failure, and finally a high mortality rate [11, 12].

Most previous studies have evaluated depression and anxiety in PLWH separately. However, the prevalence of comorbid depression and anxiety is high, ranging from 10 to 80% in PLWH according to previous studies based on different diagnostic criteria [13]. Analyzing depression and anxiety independently from each other and other covariates gives only a small picture of how these symptoms interact. More insight into the complex interconnectedness among these constructs is needed. Based on symptom network theory, generating symptom networks of multidimensional symptom experience and exploring bridge symptoms and interconnectedness between the two clusters can provide additional data, such as bridge expected influences and bridge strength. These factors have clinical implications and could lead to the development of more precise and individualized interventions [14, 15]. Previous studies have developed symptom networks in various populations, including cancer patients receiving chemotherapy, people living with HIV, and people with mental disorders, to capture complex relationships among symptoms of various chronic diseases [16–18].

Previous studies have indicated that controlling bridge symptom severity in dynamic psychopathology models may enhance intervention efficacy [19–21]. Lunansky and colleagues used the NodeIdentifyR algorithm to identify bridge symptoms that had the greatest impact on total symptom activation among three post-traumatic stress disorder symptom clusters [19]. Groen and colleagues assessed the role of bridge symptoms in dynamic psychological networks and suggested that bridge symptoms should serve as target for psychotherapeutic interventions but not for prevention strategies [20]. Castro and colleagues summarized published data and found that interventions targeting bridge symptoms were more efficient than other symptoms. However, this conclusion was supported by only moderate amounts of empirical evidence [21].

To enhance the efficacy and efficiency of psychotherapeutic approaches, it is crucial to identify the bridge symptoms. However, few studies have developed networks of multidimensional symptoms to identify bridge symptoms and evaluate interconnectedness between depression and anxiety in PLWH with comorbid depressive and anxiety disorders. It is still unclear which symptom is the bridge symptom between depression and anxiety in PLWH with comorbid depressive and anxiety disorders. Empirical evidence is needed to develop personalized and precise psychological intervention strategies specific for PLWH with comorbid depressive and anxiety disorders. Therefore, the objectives of this study were to 1) generate symptom networks of depression and anxiety in PLWH with comorbid depressive and anxiety disorders and 2) explore bridge symptoms and interconnectedness between depression and anxiety.

## Methods

### Study design and settings

We conducted a multisite, hospital-based cross-sectional study named “Living Positively with HIV/AIDS (LIPHA)” between March 2020 and November 2021. Participants were recruited from three HIV/AIDS-designated hospitals in Shanghai, Shenzhen (Guangdong Province), and Kunming (Yunnan Province), China. Shanghai and Shenzhen have more than 30,000 and 21,000 HIV cases, respectively, and 58–65% of those infections were in men who have sex with men. Kunming is the capital city of Yunnan Province and has over 12,000 cases. The prevalence of HIV in these three cities is representative of southern China in urban areas. The Institutional Review Board of Fudan University School of Nursing approved this study (IRB# TYSQ20210304). Written informed consent was obtained from all subjects prior to enrollment in the study.

### Participants

The inclusion criteria were as follows: 1) diagnosed with HIV; 2) aged 18 years or older; 3) diagnosed as having depressive and anxiety disorders by the Diagnostic and Statistical Manual of Mental Disorders, Fifth Edition (DSM-5); 3) scored ≥1 on the Hospital Anxiety and Depression Scale (HADS); and 4) provided informed consent. Participants who were unable to complete the self-rating scale due to severe comorbidities and/or cognitive impairment were excluded. Finally, a total of 2046 PLWH participated in this study. Thirty participants (1.5%) were excluded due to missing or invalid data. The remaining 2016 were included in the analysis.

### Measures

Eligible participants were asked to provide written informed consent before data collection. The survey was delivered by the members of our research team. Participants were compensated with gifts. The following sections were included in the survey.

#### Depression and anxiety

Depression and anxiety were measured by the Hospital Anxiety and Depression Scale (HADS) [22]. The HADS is a 14-item scale and includes two dimensions: depression (7 items) and anxiety (7 items). A 4-point Likert scale was used for the responses, ranging from 1 to 4. Two dimensions were scored separately. For both dimensions, a higher score indicated a more severe condition. The HADS has reported good internal consistency. The Cronbach’s α was 0.828 for the anxiety dimension and 0.859 for the depression dimension.

#### Covariates

Covariates included age (continuous), sex (male and female), marital status, education, employment status, years since HIV diagnosis, HIV status disclosure, having ART, ART regimen, prescribed ART that has neurological side effects (e.g., dolutegravir, efavirenz, and rilpivirine), CD4+ T cell count, and viral load. All covariates were collected with a standardized questionnaire.

### Data analysis

All statistical analyses were conducted using R version 1.6.4. The demographic characteristics and severity of symptoms are described using frequencies, percentages, means, and standard deviations.

#### Aim 1: characterization and interconnectedness of depression/anxiety networks

We constructed contemporaneous networks with all 14 psychological symptoms. Covariates that were significantly associated with the overall HADS score were included in the network analysis to identify the real relationships among the symptoms after controlling for confounding factors. Each node represented one symptom. Edges in the network represented the conditional independent relationships between two nodes; the thicker the edges were, the stronger the association between two nodes. We visualized the network using the *qgraph* package. The GLASSO layout was used to generate undirected association networks.

Bootstrapping methods were performed to assess the accuracy and stability of the network by using the R package *bootnet*. The accuracy was evaluated by calculating the 95% confidence intervals (CIs) of the edge weight values. We used nonparametric bootstrapping (1000 bootstrap samples) to construct CIs [23]. To identify whether the estimations of network connections and centrality for different variables differ, we performed bootstrapped difference tests between edge weights and centrality indices in the LASSO regularization of partial correlation networks based on polychoric correlation matrices [24].

#### Aim 2: analyzing the bridge between depression and anxiety

To identify the bridge symptoms that had the strongest connection with the depression and anxiety clusters, we computed the bridge expected influence. The bridge expected influence was calculated by the accumulation of the node’s edge weight. Based on the bootstrap analysis, tests for significant differences between individual nodes’ expected bridge values were conducted.

#### Aim 3: node centrality and predictability

To identify the most central symptoms, we conducted a centrality analysis with the following three centrality indices: strength, betweenness, and closeness. The strength of a symptom network is an indicator of network connectivity. A higher strength centrality means that the symptom is more likely to occur in conjunction with other symptoms. Betweenness is measured by the number of times a node acts as a bridge along the shortest path between two nodes. A node with a higher betweenness centrality has greater network influence. Closeness is indicated by the average distance (inverse distance) between one symptom and all other nodes. The shorter the path is, the greater the closeness value.

Moreover, we used the *mgm* package to identify the predictability for each node. A symptom with a high value of predictability indicated that we could control the symptom via its neighboring nodes. In contrast, if the value of predictability is low, we need to intervene in the symptom directly or look for a marker outside the network. The stability was evaluated by calculating the correlation stability coefficient of the expected impact of nodes using a case-dropping subset bootstrap (1000 bootstrap samples) [24]. The correlation stability coefficient should preferably be greater than 0.5, but at the very least it should be greater than 0.25 [25].

## Results

### Participant characteristics and prevalence and severity of symptoms

Table 1 shows the sample characteristics (*n* = 2016). The mean age of the participants was 41.20 ± 12.70 years old, ranging from 18 to 81 years old. The majority of participants were male (1806, 89.58%), single (929, 46.08%), and had a university degree or more (655, 32.49%). The median number of years since HIV infection diagnosis was 3.67 ± 3.92 (0–34), and 91.47% of these respondents (1844) had received ART. More than 51.59% of these participants were taking medications (e.g., dolutegravir, efavirenz, and rilpivirine) that have neurological side effects and could cause depressive symptoms.

**Table 1:** Characteristics of participants (*n* = 2016)

Characteristics	n (%), M ± SD (IQR)
**Age**	41.20 ± 12.70 (18–81)
**Gender**	
Male	1806 (89.58)
Female	209 (10.37)
**Education**	
Primary school or below	362 (17.96)
Secondary school	504 (25.00)
Post-secondary	495 (24.55)
University or above	655 (32.49)
**Marital status**	
Single	929 (46.08)
Married	780 (38.69)
Otherwise	307 (15.23)
**Employment**	
Employed	1708 (84.72)
Otherwise	308 (15.27)
**Year since HIV diagnosis**	3.67 ± 3.92 (0–34)
**HIV status disclosure**	
Undisclosed	801 (39.73)
Disclose to friends	346 (17.16)
Disclose to family member	698 (34.62)
**Prescribed ART (Yes)**	1844 (91.47)
**Prescribed ART that has neurological side effects**	1040 (51.59)
**CD4+ T cell count**	326.72 ± 263.09 (1–1247)
0–50	389 (19.30)
50–200	419 (20.78)
200–500	675 (33.48)
> 500	533 (26.44)
**Viral load (RNA)**	
< 500	1232 (61.11)
> 500	784 (38.89)

*ART* antiretroviral therapy

Table 2 shows the prevalence and severity of all symptoms of depression and anxiety. The most prevalent depressive symptom was “not feeling cheerful” (*n* = 1616, 80.16%), followed by “lost interest in appearance” (*n* = 1513, 75.05%). The most prevalent anxious symptoms were “not sitting at ease and feeling relaxed” (*n* = 1659, 82.29%) and “having frightened feelings like ‘butterflies’ in the stomach” (*n* = 1637, 81.20%). The most severe depressive and anxious symptoms were “lost interest in appearance” (2.61 ± 1.67) and “having frightened feelings like ‘butterflies’ in the stomach” (2.60 ± 1.34).

**Table 2:** Prevalence, severity, and predictability of all symptoms of depression and anxiety (*n* = 2016)

Symptoms	Abbreviation	Prevalence (n, %)	Mean ± SD	Predictability
Feeling tense or ‘wound up’	HADS1	1462 (72.52%)	1.89 ± 0.71	80.9%
Not enjoying the things that used to enjoy	HADS2	1274 (63.19%)	1.93 ± 0.91	83.6%
Having frightened feeling as if something awful is about to happen	HADS3	1528 (75.79%)	2.15 ± 0.86	76.2%
Not being able to laugh and see the funny side of things	HADS4	1072 (53.17%)	1.92 ± 1.00	82.3%
Worrying thoughts go through mind	HADS5	765 (37.95%)	1.56 ± 0.84	79.6%
Not feeling cheerful	HADS6	1616 (80.16%)	2.59 ± 1.05	67.1%
Not sitting at ease and feeling relaxed	HADS7	1659 (82.29%)	2.55 ± 1.11	70.3%
Feeling being slowed down	HADS8	1511 (74.95%)	2.50 ± 1.07	65.0%
Having frightened feeling like ‘butterflies’ in the stomach	HADS9	1637 (81.20%)	2.60 ± 1.34	71.7%
Lost interest in appearance	HADS10	1513 (75.05%)	2.61 ± 1.67	66.0%
Feeling restless	HADS11	1310 (64.98%)	1.87 ± 0.84	86.1%
Not looking forward with enjoyment to things	HADS12	1234 (61.21%)	1.87 ± 0.85	85.4%
Getting sudden feelings of panic	HADS13	1345 (66.72%)	1.86 ± 0.76	75.5%
Not enjoying a good book or radio or TV program	HADS14	1077 (53.42%)	1.87 ± 1.04	86.2%

### Characterization and interconnectedness of symptom networks

**Fig. 1:**
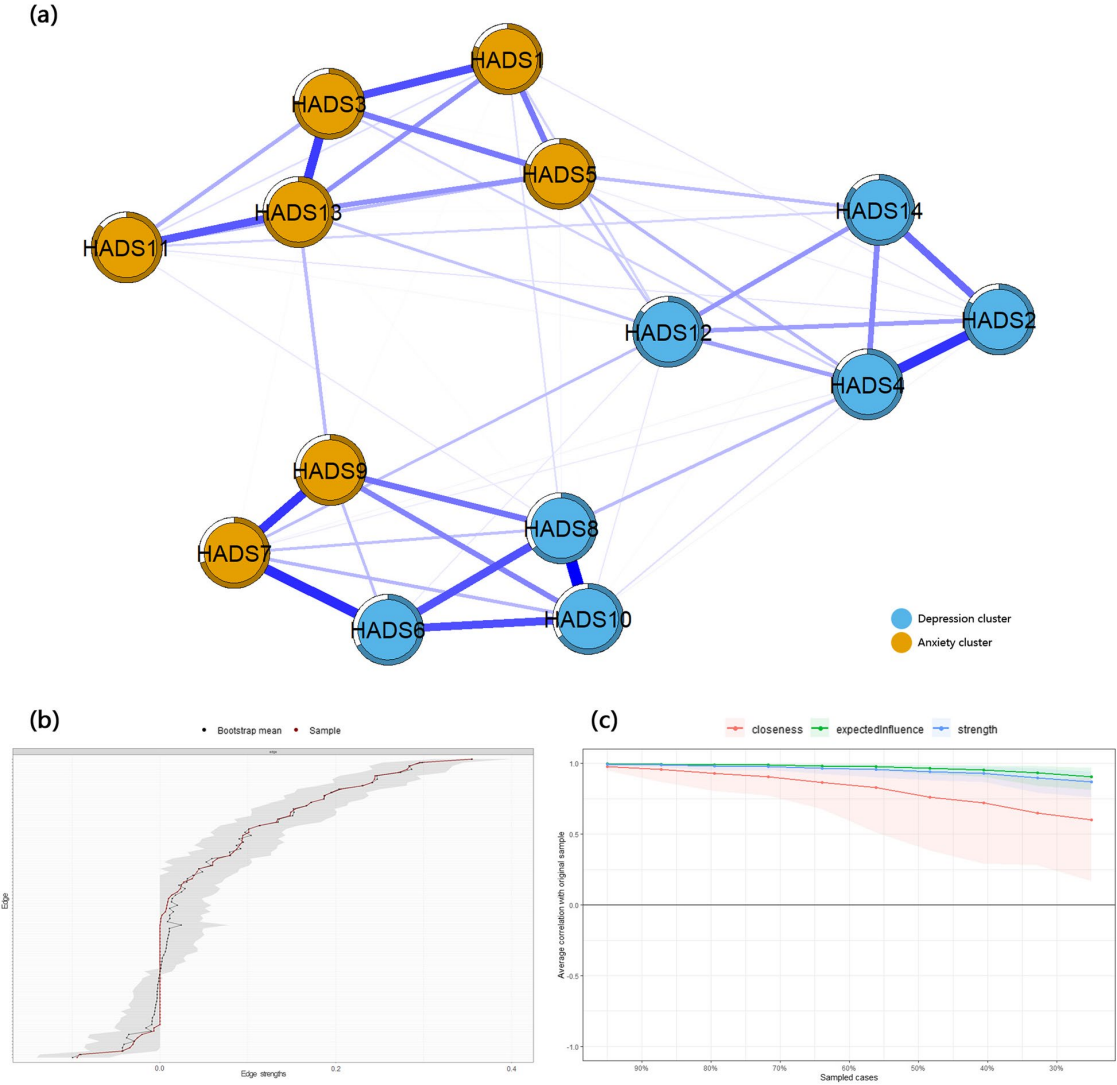
Characterization and interconnectedness of symptom networks. **a** Symptom network; **b** Accuracy of the symptom network; **c** Stability of the symptom network. HADS1: feeling tense or wound up; HADS2: not enjoying the things that used to enjoy; HADS3: having frightened feeling as if something awful is about to happen; HAD4: not being able to laugh and see the funny side of things; HAD5: worrying thoughts go through mind; HADS6: mot feeling cheerful; HADS7: not sitting at ease and feeling relaxed; HADS8: feeling being slowed down; HADS9: having frightened feeling like ‘butterflies’ in the stomach; HADS10: lost interest in appearance; HADS11: feeling restless; HADS12: not looking forward with enjoyment to things; HADS13: getting sudden feelings of panic; HADS14: not enjoying a good book or radio or TV program

**Fig. 2:**
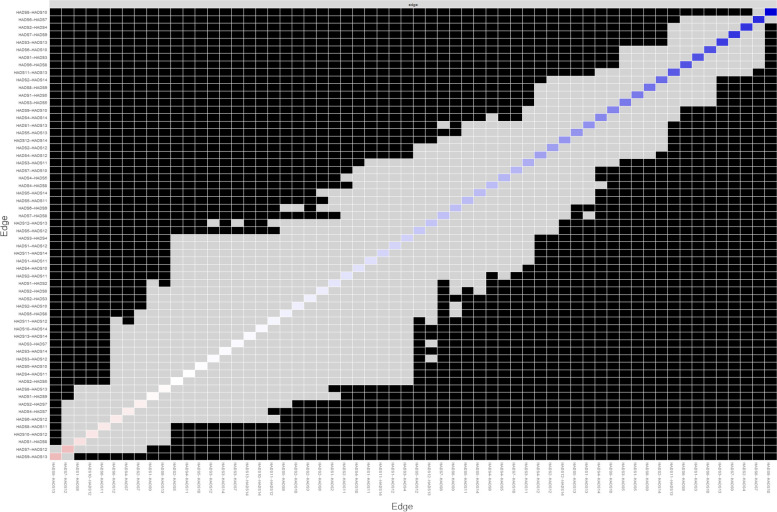
Interconnectedness of nodes. HADS1: feeling tense or wound up; HADS2: not enjoying the things that used to enjoy; HADS3: having frightened feeling as if something awful is about to happen; HAD4: not being able to laugh and see the funny side of things; HAD5: worrying thoughts go through mind; HADS6: mot feeling cheerful; HADS7: not sitting at ease and feeling relaxed; HADS8: feeling being slowed down; HADS9: having frightened feeling like ‘butterflies’ in the stomach; HADS10: lost interest in appearance; HADS11: feeling restless; HADS12: not looking forward with enjoyment to things; HADS13: getting sudden feelings of panic; HADS14: not enjoying a good book or radio or TV program

Figure 1(a) shows the network of depressive and anxious symptoms after controlling covariates (age: *β* = − 0.164, *P* < 0.001; having a university degree or more: *β* = − 0.103, *P* = 0.004; and employed *β* = − 0.092, *P* = 0.008). Figure 1(b) shows the bootstrap analysis results of the edge weights. The bootstrapped CIs were small, which showed good accuracy of the network. For the subset bootstrap in Fig. 1(c), the correlation stability coefficient was 0.75, suggesting that the network remained stable. In Fig. 2, we found that the strongest edge weights, HADS8 (feeling being slowed down) and HADS10 (lost interest in appearance) (r = 0.36 ± 0.02), were significantly different from the other edge weights. HADS6 (not feeling cheerful) and HADS7 (not sitting at ease and feeling relaxed) were the strongest edges across the depression and anxiety clusters (r = 0.30 ± 0.02).

### Bridge centrality

**Fig. 3:**
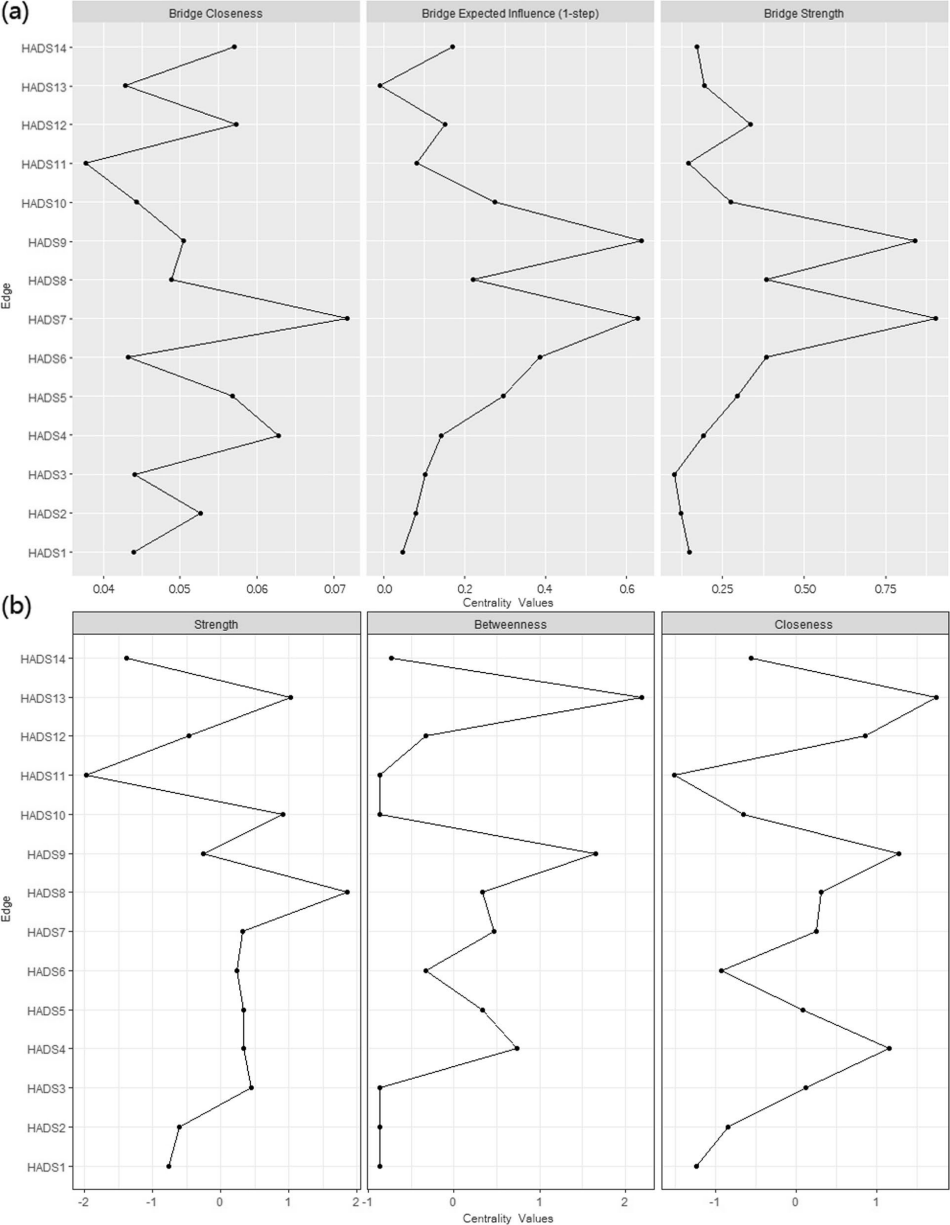
Node centrality. **a** Bridge node centrality; **b** Node centrality. HADS1: feeling tense or wound up; HADS2: not enjoying the things that used to enjoy; HADS3: having frightened feeling as if something awful is about to happen; HAD4: not being able to laugh and see the funny side of things; HAD5: worrying thoughts go through mind; HADS6: mot feeling cheerful; HADS7: not sitting at ease and feeling relaxed; HADS8: feeling being slowed down; HADS9: having frightened feeling like ‘butterflies’ in the stomach; HADS10: lost interest in appearance; HADS11: feeling restless; HADS12: not looking forward with enjoyment to things; HADS13: getting sudden feelings of panic; HADS14: not enjoying a good book or radio or TV program

Figure 3(a) shows the centrality of bridge symptoms of the two clusters. In the anxiety cluster, HADS7 (not sitting at ease and feeling relaxed) had the highest bridge expected influence and strength (*r*_*bridge expected influence*_ = 0.628, *r*_*strength*_ = 0.903), followed by HADS9 (having frightened feeling like ‘butterflies’ in the stomach) (*r*_*bridge expected influence*_ = 0.638, *r*_*strength*_ = 0.840). In the depression cluster, HADS 6 (not feeling cheerful) was identified as a bridge symptom with a high value of bridge expected influence (*r*_*bridge expected influence*_ = 0.385).

### Node centrality and predictability

Figure 3(b) presented three centrality indices. Among depressive symptoms, HADS8 had the largest values for node centrality (*r*_*strength*_ = 1.101, *r*_*betweenness*_ = 38.000, *r*_*closeness*_ = 0.006). Among anxious symptoms, HADS13 had the largest values for node centrality (*r*_*strength*_ = 0.999, *r*_*betweenness*_ = 46.000, *r*_*closeness*_ = 0.006). Predictability is presented as a circle around a node in Fig. 1(a). Table 2 shows that the node predictability values ranged from 65.0 to 86.2%. HADS11 (feeling restless), HADS12 (not looking forward with enjoyment to things), and HADS14 (not enjoying a good book or radio or TV program) had the highest predictability, showing that 86.1, 85.4, and 86.2% of their variance can be explained by their neighbors.

## Discussion

This is the first study that developed a symptom network to explore bridge symptoms and interconnectedness between depression and anxiety in a large sample of PLWH with comorbid depressive and anxiety disorders. We found that “not feeling cheerful” and “not feeling relaxed” were two bridge symptoms between the depression and anxiety clusters. The network also showed stronger correlations between “not feeling cheerful” and “not feeling relaxed” and “feeling slowed down” and “lost interest in appearance” compared to other relationships. In the depression and anxiety subnetworks, “feeling slowed down” and “panic” were the core symptoms.

We found that “not feeling cheerful” and “not feeling relaxed” were the bridge symptoms in the depression and anxiety clusters, which indicated that these symptoms could serve as bridges between clusters. In complex networks, bridge nodes are essential for calculating the co-occurrence of clusters [26]. This result is noteworthy because a large number of PLWH may have both depressive and anxious symptoms at the same time due to stigma, intolerance of uncertainty, fear of death, and medications. Furthermore, previous studies reported that depressive and anxious symptoms could degenerate to a certain extent and finally lead to the comorbidity of depression and anxiety disorders [27, 28]. It is accepted that PLWH may have one or a couple of negative emotions that are regarded as a defense mechanism protecting them from stress and trauma [29]. However, too many co-occurring symptoms may increase the connectivity of the network, which is the prime property for detecting changes in prognosis [17]. Therefore, interventions targeting bridge symptoms might be more efficient and precise than overall psychotherapeutic interventions in altering the interconnectedness between the subnetworks of depression and anxiety [30].

To enhance the levels of perceived cheerfulness and relaxation, multiple psychotherapeutic strategies that are currently used to treat depressive and anxiety disorders can be applied. Psychotherapy encompasses many aspects and approaches, and various treatments may focus on specific symptoms or age ranges to achieve better therapeutic outcomes. Previous research has suggested that cognitive behavioral therapy may be most effective in reducing symptoms related to “worry” and that positive thought-based therapies may be most effective in reducing the inability to relax [31, 32]. The Adaptive Information Processing model and the therapeutic hierarchy have been shown to reduce worry-related symptoms [33]. The introduction of computer games, a play therapy strategy, has also been found to relax children and adolescents [34].

Compared to these psychological therapies, positive psychology interventions such as meaning-oriented interventions, strength-building measures, optimistic interventions, kindness boosters, and multicomponent positive psychology interventions are more suited to target “not feeling cheerful” and “not feeling relaxed”. Thus, positive psychology could be used to enhance PLWH’s cheerfulness, psychological wellbeing, and positive cognitions and emotions [35, 36]. Regarding the efficacy and effectiveness of positive psychology interventions, Hendriks and colleagues’ systematic review included 50 randomized controlled trials and found that positive psychology interventions had only a small effect on depression and anxiety [37]. Previous studies further showed that the effectiveness of positive psychological intervention could vary depending on PLWH’s age, race, education, and stress level [38, 39]. Although positive psychological interventions reported a low level of effectiveness in decreasing the severity of anxiety and depression, our study provided a hypothesis based on empirical data: that enhancing cheerfulness can help cut off the bridge between depression and anxiety clusters. Psychotherapeutic interventions that may potentially improve cheerfulness can be included to help decrease the interaction between depression and anxiety. Future studies should examine this hypothesis and further identify the inter-cluster and trans-cluster functions of bridge symptoms, which may offer empirical evidence for developing frameworks for real-world clinical interventions. In addition, longitudinal data are needed to explore PLWH’s dynamic networks, which can provide causality information on how bridge symptoms affect both depression and anxiety clusters.

This study also found that there were strong correlations among “cheerfulness”, “relaxation”, “being slowed down”, “lost interest in appearance”, and “butterfly feelings”. These five symptoms comprised a “bridge section” in the whole network, indicating that symptoms in the bridge section were more engaged than other symptoms in aggregating depressive and anxious symptoms and preserving the integrity of the entire network of interactions [40]. Although this study analyzed cross-sectional data and could not identify the true causality among symptoms, the predictability indicator could provide a hypothesis of causality interference in complex networks [14]. The predictability of five symptoms in the bridge section was lower than that of other symptoms. These results indicated that the changes in symptoms in the bridge section might be independent of all connected symptoms and that recommended therapy should intervene with these symptoms directly [41]. Consistent with this discovery, previous studies also found that symptoms with high predictability are only weakly connected with the causal effect of symptoms in the network [42, 43]. Therefore, in addition to the two main bridge symptoms (“not feeling cheerful” and “not feeling relaxed”), symptoms in the bridge section should also be considered as targets in psychotherapeutic interventions. Healthcare professionals should also be cautious when PLWH report high severity symptoms in the bridges section.

## Limitations

Although this is the first study to develop a symptom network to explore bridge symptoms in PLWH with comorbid depressive and anxiety disorders, it has some limitations. First, our study had a cross-sectional design. Therefore, we could not establish causation among symptoms. Our study can only provide potential hypothesis for future longitudinal and intervention study. Second, this study recruited PLWH with comorbid depressive and anxiety disorders at treatment follow-ups in the outpatient department. The HADS scores for anxiety and depression were relatively low compared to those of inpatient PLWH. Third, our study did not analysis the influence of sociodemographic factors on the findings. In this study, the included PLWH were from urban settings. It remains unclear whether PLWH in rural areas differ. Future studies are needed to assess the external validity of our results in various settings. Finally, survivorship bias may have influenced the results, as PLWH without follow-up were not included in this study. Therefore, the prevalence, symptom severity, and centrality indices may have been underestimated.

## Conclusion

Our study explored bridge symptoms and interconnectedness between depression and anxiety in PLWH. We found that “not feeling cheerful” and “not feeling relaxed” were two bridge symptoms between the depression and anxiety clusters. Future studies should utilize a longitudinal design to explore dynamic networks in PLWH, which can determine causal relationships, such as how bridge symptoms affect both the depression and anxiety clusters. We recommend that healthcare professionals should take note when PLWH report severe bridge symptoms. To enhance the levels of perceived cheerfulness and relaxation, positive psychology interventions such as meaning-oriented interventions, strength-building measures, optimistic interventions, kindness boosters, and multicomponent positive psychology interventions could be used to target symptoms such as “not feeling cheerful” and “not feeling relaxed”.

## Data Availability

The datasets generated and/or analyzed during the current study are not publicly available due to individual data from people living with HIV should be protected according to hospitals’ regulations, but are available from the corresponding author on reasonable request.

## Abbreviations

AIDS: Acquired Immune Deficiency Syndrome
ART: Antiretroviral therapy
HAD: Hospital Anxiety and Depression Scale
HIV: Human Immunodeficiency Virus
PLWH: Patients living with HIV

## References

[1] World Health Organization. News room Fact sheet HIV/AIDS https://www.who.int/news-room/fact-sheets/detail/hiv-aids (accessed December 16, 2021).

[2] Deeks SG, Lewin SR, Havlir DV (2013). The end of AIDS: HIV infection as a chronic disease. Lancet..

[3] Remien RH, Stirratt MJ, Nguyen N, Robbins RN, Pala AN, Mellins CA (2019). Mental health and HIV/AIDS: the need for an integrated response. AIDS..

[4] Rooks-Peck CR, Adegbite AH, Wichser ME, Ramshaw R, Mullins MM, Higa D, Sipe TA (2018). Prevention research synthesis project. Mental health and retention in HIV care: a systematic review and meta-analysis. Health Psychol.

[5] Wang T, Fu H, Kaminga AC, Li Z, Guo G, Chen L, Li Q (2018). Prevalence of depression or depressive symptoms among people living with HIV/AIDS in China: a systematic review and meta-analysis. BMC Psychiatry.

[6] Rezaei S, Ahmadi S, Rahmati J, Hosseinifard H, Dehnad A, Aryankhesal A, Shabaninejad H, Ghasemyani S, Alihosseini S, Bragazzi NL, Raoofi S, Kiaee ZM, Ghashghaee A (2019). Global prevalence of depression in HIV/AIDS: a systematic review and meta-analysis. BMJ Support Palliat Care.

[7] Nakimuli-Mpungu E, Musisi S, Smith CM, Von Isenburg M, Akimana B, Shakarishvili A, Nachega JB, Mills EJ, Chibanda D, Ribeiro M, Williams VA, Joska JA (2021). Mental health interventions for persons living with HIV in low- and middle-income countries: a systematic review. J Int AIDS Soc.

[8] Chaponda M, Aldhouse N, Kroes M, Wild L, Robinson C, Smith A (2018). Systematic review of the prevalence of psychiatric illness and sleep disturbance as co-morbidities of HIV infection in the UK. Int J STD AIDS.

[9] Zhu Z, Hu Y, Xing W, Guo M, Zhao R, Han S, Wu B (2019). Identifying symptom clusters among people living with HIV on antiretroviral therapy in China: a network analysis. J Pain Symptom Manag.

[10] Kessler RC, Chiu WT, Demler O, Walters EE (2005). Prevalence, severity, and comorbidity of 12-month DSM-IV disorders in the National Comorbidity Survey Replication. Arch Gen Psychiatry.

[11] Too EK, Abubakar A, Nasambu C, Koot HM, Cuijpers P, Newton CR, Nyongesa MK (2021). Prevalence and factors associated with common mental disorders in young people living with HIV in sub-Saharan Africa: a systematic review. J Int AIDS Soc.

[12] Deshmukh NN, Borkar AM, Deshmukh JS (2017). Depression and its associated factors among people living with HIV/AIDS: can it affect their quality of life?. J Family Med Prim Care.

[13] Camara A, Sow MS, Touré A, Sako FB, Camara I, Soumaoro K, Delamou A, Doukouré M (2020). Anxiety and depression among HIV patients of the infectious disease department of Conakry University Hospital in 2018. Epidemiol Infect.

[14] Zhu Z, Xing W, Hu Y, Wu B, So W. Paradigm shift: moving from symptom clusters to symptom networks. Asia Pac J Oncol Nurs. 2022. 10.1016/j.apjon.2021.12.001 PMC9072174 35528791

[15] Fried EI, Boschloo L, van Borkulo CD, Schoevers RA, Romeijn JW, Wichers M, de Jonge P, Nesse RM, Tuerlinckx F, Borsboom D (2015). Commentary: "consistent superiority of selective serotonin reuptake inhibitors over placebo in reducing depressed mood in patients with major depression". Front Psychiatry..

[16] Rha SY, Lee J (2021). Stable symptom clusters and evolving symptom networks in relation to chemotherapy cycles. J Pain Symptom Manag.

[17] Schweren L, van Borkulo CD, Fried E, Goodyer IM (2018). Assessment of symptom network density as a prognostic marker of treatment response in adolescent depression. JAMA Psychiatry.

[18] Zhu Z, Wen H, Yang Z, Han S, Fu Y, Zhang L, Hu Y, Wu B (2021). Evolving symptom networks in relation to HIV-positive duration among people living with HIV: a network analysis. Int J Infect Dis.

[19] Lunansky G, Naberman J, van Borkulo CD, Chen C, Wang L, Borsboom D (2021). Intervening on psychopathology networks: evaluating intervention targets through simulations [published online ahead of print, 2021 Nov 16]. Methods..

[20] Groen RN, Ryan O, Wigman JTW, et al. Comorbidity between depression and anxiety: assessing the role of bridge mental states in dynamic psychological networks. BMC Med. 2020;18(1):308. Published 2020 Sep 29. doi:10.1186/s12916-020-01738-z 10.1186/s12916-020-01738-z PMC7523307 32988400

[21] Castro D, Ferreira F, de Castro I, Rodrigues AR, Correia M, Ribeiro J, Ferreira TB (2019). The differential role of central and bridge symptoms in deactivating psychopathological networks. Front Psychol.

[22] Zigmond AS, Snaith RP (1983). The hospital anxiety and depression scale. Acta Psychiatr Scand.

[23] Efron B (2000). The bootstrap and modern statistics. J Am Stat Assoc.

[24] Epskamp S, Borsboom D, Fried EI (2018). Estimating psychological networks and their accuracy: a tutorial paper. Behav Res Methods.

[25] Armour C, Fried EI, Deserno MK, Tsai J, Pietrzak RH (2017). A network analysis of DSM-5 posttraumatic stress disorder symptoms and correlates in U.S. military veterans. J Anxiety Disord.

[26] Ahmed A, Saqlain M, Umair MM, Hashmi FK, Saeed H, Amer M, Blebil AQ, Dujaili JA (2021). Stigma, social support, illicit drug use, and other predictors of anxiety and depression among HIV/AIDS patients in Pakistan: a cross-sectional study. Front Public Health.

[27] Duko B, Toma A, Asnake S, Abraham Y (2019). Depression, anxiety and their correlates among patients with HIV in South Ethiopia: an institution-based cross-sectional study. Front Psychiatry..

[28] Smith KE, Mason TB, Crosby RD, Cao L, Leonard RC, Wetterneck CT, Smith BER, Farrell NR, Riemann BC, Wonderlich SA, Moessner M (2019). A comparative network analysis of eating disorder psychopathology and co-occurring depression and anxiety symptoms before and after treatment. Psychol Med.

[29] Cramer P (2008). Seven pillars of defense mechanism theory. Soc Personal Psychol Compass.

[30] Kaiser T, Herzog P, Voderholzer U, Brakemeier EL (2021). Unraveling the comorbidity of depression and anxiety in a large inpatient sample: network analysis to examine bridge symptoms. Depress Anxiety.

[31] David D, Cristea I, Hofmann SG (2018). Why cognitive behavioral therapy is the current gold standard of psychotherapy. Front Psychiatry.

[32] Goldberg SB, Tucker RP, Greene PA (2018). Mindfulness-based interventions for psychiatric disorders: a systematic review and meta-analysis. Clin Psychol Rev.

[33] Gierus J (2020). Information processing and decision-making in pathological worriers and their potential role in mechanisms of generalized anxiety disorder. Adv Cogn Psychol.

[34] Zayeni D, Raynaud JP, Revet A (2020). Therapeutic and preventive use of video games in child and adolescent psychiatry: a systematic review. Frontiers in psychiatry.

[35] Carr A, Cullen K, Keeney C, Canning C, Mooney O, Chinseallaigh E, O’Dowd A (2021). Effectiveness of positive psychology interventions: a systematic review and meta-analysis. J Posit Psychol.

[36] Bolier L, Haverman M, Westerhof GJ, Riper H, Smit F, Bohlmeijer E (2013). Positive psychology interventions: a meta-analysis of randomized controlled studies. BMC Public Health.

[37] Hendriks T, Schotanus-Dijkstra M, Hassankhan A, De Jong J, Bohlmeijer E (2020). The efficacy of multi-component positive psychology interventions: a systematic review and Meta-analysis of randomized controlled trials. J Happiness Stud.

[38] Addington EL, Cheung EO, Moskowitz JT (2020). Who is most likely to benefit from a positive psychological intervention? Moderator analyses from a randomized trial in people newly diagnosed with HIV. J Posit Psychol.

[39] Antoine P, Dauvier B, Andreotti E, Congard A (2018). Individual differences in the effects of a positive psychology intervention: applied psychology. Pers Individ Dif.

[40] Groen RN, Ryan O, Wigman JTW, Riese H, Penninx BWJH, Giltay EJ, Wichers M, Hartman CA (2020). Comorbidity between depression and anxiety: assessing the role of bridge mental states in dynamic psychological networks. BMC Med.

[41] Haslbeck JMB, Waldorp LJ (2018). How well do network models predict observations? On the importance of predictability in network models. Behav Res Methods.

[42] Rodebaugh TL, Tonge NA, Piccirillo ML, Fried E, Horenstein A, Morrison AS, Goldin P, Gross JJ, Lim MH, Fernandez KC, Blanco C, Schneier FR, Bogdan R, Thompson RJ, Heimberg RG (2018). Does centrality in a cross-sectional network suggest intervention targets for social anxiety disorder?. J Consult Clin Psychol.

[43] Dablander F, Hinne M (2019). Node centrality measures are a poor substitute for causal inference. Sci Rep.

